# Factors Affecting COVID-19 Vaccination among the General Population in Saudi Arabia

**DOI:** 10.3390/healthcare9091218

**Published:** 2021-09-16

**Authors:** Khalid Al-Mansour, Saad Alyahya, Fouad AbuGazalah, Khaled Alabdulkareem

**Affiliations:** 1 Department of Social Studies, College of Arts, King Saud University, Riyadh 11451, Saudi Arabia; 2 General Administration for Primary Health Centers, Ministry of Health, Riyadh 12628, Saudi Arabia; 3 Nudge Unite, Ministry of Health, Riyadh 12628, Saudi Arabia; Salyahya@moh.gov.sa; 4 Health Holding Company, Riyadh 12628, Saudi Arabia; FaboGazalah@moh.gov.sa; 5 Deputyship for Primary Health Care, Ministry of Health, Riyadh 12628, Saudi Arabia; Khalabdulkarim@moh.gov.sa; 6 Department of Family Medicine, College of Medicine, Al-Imam Mohammad Ibn Saud Islamic University, Riyadh 11564, Saudi Arabia

**Keywords:** COVID-19 vaccine, general population, knowledge, concerns, Saudi Arabia

## Abstract

Vaccine refusal or hesitancy is one of the significant issues that can have an adverse impact on people’s health and their countries’ economy. Additionally, vaccine acceptance or refusal could have a decisive role in controlling the COVID-19 pandemic. This study aims to investigate the factors affecting COVID-19 vaccine refusal and hesitancy among the general population in Saudi Arabia. The method is a cross-sectional survey using an online questionnaire, and data were collected from 1935 participants between 18 February 2021 and 1 April 2021. Out of 1935 participants aged ≥18 years and residing in Saudi Arabia, 46.9% reported their intention to receive the COVID-19 vaccine, 22.4% had received the vaccine, 9.5% did not intend to receive the vaccine, and 21.2% had not made their decision. In the multinomial logistic regression models, vaccine refusal was associated with age (OR: 0.961), nationality (OR: 0.182), monthly income of more than SAR 18,000 (OR: 2.325), chronic diseases (OR: 0.521), knowledge about the vaccine (OR: 0.937), and concerns about the vaccine (OR: 1.5). The hesitancy was associated with age (OR: 0.977), nationality (OR: 0.231), monthly income between SAR 6000 to 12,000 (OR: 0.607), chronic diseases (OR: 0.640), knowledge about the vaccine (OR: 0.907), and concerns about the vaccine (OR: 1.3). The main concerns about the vaccine were “COVID-19 vaccines are not tested enough on people”, “drug companies are interested in COVID-19 vaccine sales only”, and “COVID-19 vaccines have serious adverse effects”. Awareness programs and vaccination campaigns should consider people’s concerns and correct their misinformation.

## 1. Introduction

A newly detected virus, known as severe acute respiratory syndrome coronavirus-2 (SARS-CoV-2), has been shown to be the cause of the novel coronavirus disease of 2019 (COVID-19), with manifestations ranging from asymptomatic or mild respiratory symptoms to respiratory failure and death [1]. In March 2020, after spreading to more than 110 countries, the World Health Organization declared COVID-19 a pandemic [2]. As of 28 March 2021, there have been 126,697,603 confirmed cases of COVID-19, including 2,776,157 deaths worldwide [3]. In addition to its health and psychological consequences, the COVID-19 pandemic has resulted in an economic crisis associated with setting lockdowns, closing businesses, and suspending travel [4,5,6,7].

To hinder the spread of COVID-19, protective behaviors are needed, and high vaccination coverage is thought to be the key protective behavior globally [8]. To date, a few COVID-19 vaccines have been shown to be effective and safe; many pharmaceutical companies, in cooperation with governments, have received final approvals to distribute their COVID-19 vaccines, and most countries started vaccination programs [8,9]. The intention to receive COVID-19 vaccines has varied significantly across countries: 27.7% in Congo [10], 40.0% in Hong Kong [11], 61.1% in Israel [12], 77.6% in France [13], 91.3% in China [14], and 93.3% in Indonesia [15].

Saudi Arabia, on human and economic levels, is among the countries most affected by the COVID-19 pandemic in the Eastern Mediterranean region [16,17]. Therefore, the Saudi Government has collaborated with many vaccine manufacturers and set plans to vaccinate the entire population against COVID-19, starting from December 2020 [18,19]. Two recent studies investigated the COVID-19 vaccine acceptance rates among residents of Saudi Arabia before the start of the national COVID-19 vaccination campaign, and both studies reached contradicting findings: 64% and 48% [20,21]. However, the intention to receive or refuse the vaccines before their production had previously been shown to be different when the vaccine became available compared with the time before providing the vaccines [22]. Additionally, both studies did not comprehensively investigate knowledge and concerns about COVID-19 vaccines.

With vaccine development and distribution underway, it becomes imperative to know whether people in Saudi Arabia are going to receive the COVID-19 vaccine and what factors can determine their decision. It is also important to unveil the concerns of people that may prevent them from getting vaccinated. We, therefore, conducted this cross-sectional study to figure out the risk factors associated with refusal and hesitancy to receive COVID-19 vaccines among people living in Saudi Arabia and detect the people’s knowledge and concerns that may represent a challenge to the national COVID-19 vaccination campaign.

## 2. Materials and Methods

### 2.1. Study Design, Study Population, and Setting

People residing in Saudi Arabia were invited to participate in this cross-sectional study during the period between 18 February to 1 April 2021. Because of the social distancing measurements in the country, associated with the second wave of the COVID-19 pandemic, we used a non-probability snowball sampling procedure to recruit participants. Our eligibility criteria included people living in Saudi Arabia and age ≥18 years old.

The smallest sample size was calculated using Epi-Info version 7 StatCalc (CDC, Atlanta, GA, USA,) [23], which is available from the Centers for Disease Control (CDCs) and the WHO. The following criteria were adopted in the calculation: population size of 999,999, COVID-19 vaccine acceptance rate of 50%, a confidence level of 95%, and a margin of error of 5%. The minimum sample size was found to be 384 participants, which is supported by the required minimum sample size recommended by the Krejcie and Morgan sampling table [24]. However, we aimed to have more than double the minimum sample size to obtain statistical power and handle the statistical analysis in case of a low response rate.

### 2.2. Data Collection

We designed a semi-structured online survey using SurveyMonkey before sharing the link to the survey to several Facebook and Twitter groups hosting internet users from Saudi Arabia. In this snowball sampling, respondents were asked to forward the survey to their contacts and ask their contacts to forward it to their contacts and post the questionnaire in their account on Facebook and Twitter. The researcher tried to ask people from different age groups, gender, and regions to participate and distribute the survey to all of their contacts. The survey was composed of two sections. The first section included questions about participants’ sociodemographic characteristics, including sex (male or female), age in years, education (secondary school, diploma, university graduate, or post-graduate), income (<6000, 6000–12,000, 12,001–18,000, 18,001–24,000, or >24,000 Saudi Riyal/month (1 Saudi Riyal = 0.27 US Dollar)), nationality (Saudi or non-Saudi), history of COVID-19 infection (yes or no), history of chronic diseases (yes or no), and history of allergies (yes or no). The second section included a question about the intention to receive the COVID-19 vaccine: “Do you intend to receive the COVID-19 vaccine?”, and the responses were “I have received the vaccine”, “I intend to receive the vaccine”, “I do not intend to receive the vaccine”, and “not sure”. It also included six statements about COVID-19 vaccine knowledge and five statements about COVID-19 vaccine concerns. The knowledge statements were constructed based on a previous study [25], as follows: “I know people who are not allowed to receive COVID-19 vaccines”, “I know the possible side effects of COVID-19 vaccines”, “I know the steps that have been taken to test the efficacy of COVID-19 vaccines”, “I know the steps that have been taken to test the safety of COVID-19 vaccines”, “I know the opinion of scientists about COVID-19 vaccines”, and “I know the opinion of trusted health organizations about COVID-19 vaccines”. The concern statements were constructed based on previous studies [12,26], as follows: “COVID-19 vaccines have serious adverse effects”, “Drug companies are interested in COVID-19 vaccine sales only”, “The healthcare system is not trustworthy regarding COVID-19 vaccines”, “COVID-19 vaccines are ineffective”, and “COVID-19 vaccines are not tested enough on people”. Respondents had to express whether they agreed with the knowledge and concern statements on a Likert scale from one to five, with a total score of 5–30 for knowledge and 5–25 for concern. Higher scores indicated higher levels of knowledge and concern.

### 2.3. Ethical Considerations

Approval was received from the Central Institutional Review Board of the Ministry of Health in Saudi Arabia. The study adhered to the principles of the Declaration of Helsinki. The first page of the questionnaire included full details of the study. To be included in the study, respondents had to select “I agree to participate” before participation and “submit response” at the end of the survey. 

### 2.4. Statistical Analysis

Frequencies and percentages were used to describe the sociodemographic characteristics of the participants, their vaccine refusal and hesitancy rates, and vaccine knowledge and concerns. Multinomial logistic regression analyses were used to compute odds ratios (ORs) and corresponding 95% confidence intervals (CIs) of different factors associated with COVID-19 vaccine refusal and hesitancy. The dependent variable was recoded into three groups—refusal, hesitancy, and acceptance—after adding those who were already vaccinated to those who would accept the vaccination. Data analysis was conducted using Statistical Package for Social Science (SPSS), IBM SPSS Statistics for Windows, Version 23.0.

## 3. Results

Out of 1935 participants (mean age ± standard deviation = 36.6 ± 11.2), 47.8% were men, 73.2% were university graduates or had post-graduate degrees, 63.4% were Saudi citizens, 14.3% had a positive history of chronic diseases, 10.9% had a positive history of allergies, and 15.7% had a positive history of COVID-19 infection (Table 1).

More than half of the participants agreed with the statements that assessed their knowledge of the COVID-19 vaccine. For example, 52.8% agreed and 11.0% strongly agreed with the statement “I know the opinion of trusted health organizations about COVID-19 vaccines”; 48.5% agreed and 10.6% strongly agreed with the statement “I know the opinion of scientists about COVID-19 vaccines” (Table 2).

The main concerns of the participants were the following: “COVID-19 vaccines are not tested enough on people” (25.8% agreed and 10.1% strongly agreed), “drug companies are interested in COVID-19 vaccine sales only” (18.5% agreed and 7.8% strongly agreed), and “COVID-19 vaccines have serious adverse effects” (17.8% agreed and 6.7% strongly agreed) (Table 3).

**Figure 1:**
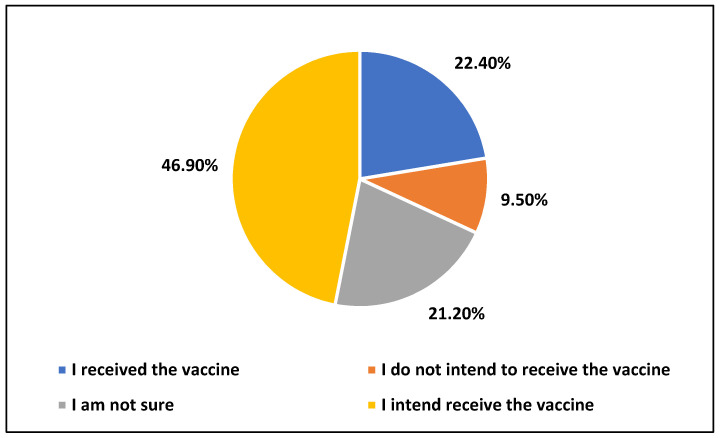
Vaccination status of participants.

Overall, 46.9% of participants reported their intention to receive the vaccine, 22.4% said that they had received the vaccine, 9.5% did not intend to receive the vaccine, and 21.2% were not sure (Figure 1).

The result of multinomial logistic regression showed that the model is significant (−2 log likelihood = 2377.175; χ^2^ (28) = 742.96, *p* < 0.001). The Nagelkerke R^2^ value was 0.398 and suggests that the predictors contributed to 39.8% of the refusal and hesitancy to uptake the COVID-19 vaccine. According to the result in Table 4, the following control variables are significant predictors for refusal: age (OR = 0.961, *p* < 0.010), nationality (OR = 0.182, *p* < 0.001), monthly income more than SAR 18,000 (OR = 2.325, *p* < 0.05), and having chronic health conditions (OR = 0.521, *p* < 0.05). Additionally, the following dependent variables are significant predictors for refusal: concerns (OR = 1.503, *p* < 0.001) and knowledge (OR = 0.937, *p* < 0.010). The following variables are not significant: gender, marital status, bachelor’s degree, graduate degree, monthly income between SAR 6000 and 12,000, monthly income between SAR 12,001 and 18,000, having allergies, and having had COVID-19.

Based on the results in Table 4. the following control variables are significant predictors for hesitancy: age (OR = 0.977, *p* < 0.05), nationality (OR = 0.231, *p* < 0.001), monthly income between SAR 6000 and 12,000 (OR = 0.607, *p* < 0.01), and having chronic health conditions (OR = 0.640, *p* < 0.05). Additionally, the following dependent variables are significant predictors for hesitancy: concerns (OR = 1.321, *p* < 0.001) and knowledge (OR = 0.907, *p* < 0.001). The following variables are not significant: gender, marital status, bachelor’s degree, graduate degree, monthly income between SAR 12,001 and 18,000, monthly income more than SAR 18,000, having allergies, and having had COVID-19.

## 4. Discussion

COVID-19 vaccine refusal and hesitancy represent a major challenge to global efforts to control the COVID-19 pandemic [27]. In addition to estimating the prevalence of COVID-19 vaccine refusal and hesitancy, this study provides a sociodemographic profile of people who are refusing or hesitant about the vaccines.

We detected that 69.3% of participants, aged ≥18 years and residing in Saudi Arabia, either received the COVID-19 vaccine or had an intention to receive it, 9.5% did not intend to receive it, and 21.2% were not sure. The COVID-19 vaccine acceptance rate in this study was 5% and 21% higher than those documented in two previous online studies (conducted in November and December 2020 on people living in Saudi Arabia who were recruited using the same sampling approaches adopted in the current study) [20,21]. The significant increase in the COVID-19 vaccine acceptance rate over the past few months is due to the positive effects of campaigns launched by the Ministry of Health in Saudi Arabia to encourage people to receive the vaccine, the initiatives taken by many top officials to receive the vaccines in front of cameras, and the provision of vaccines for free.

This study also unveiled several risk factors for vaccine refusal and hesitancy. For example, younger participants were more likely to express refusal or hesitancy than older participants. The inverse relationship between age and vaccine refusal, due to the high-risk perception among older people, comes in line with previous literature [28]. However, the sex of participants did not affect their refusal or hesitancy. While some studies have shown higher vaccine acceptance among men, other studies have shown that men were more reluctant to seek medical care [29,30] and women were more likely to practice preventive behaviors [31,32]. Thus, the impact of sex on vaccine acceptance is still a matter of debate.

Contrary to our expectations, people with chronic diseases were more likely to report vaccine refusal and hesitancy. This is despite the fact that chronic diseases are considered significant risk factors for morbidity and mortality among people with COVID-19 [33,34]. Since chronic diseases were self-reported, the possibility of misclassification bias cannot be excluded; hence, this finding needs to be further investigated with more details.

Concerns about COVID-19 vaccines were significantly associated with higher vaccine refusal and hesitancy rates. Most of these concerns were about the safety and effectiveness of the vaccines and a lack of trust in drug companies. On the other hand, having good knowledge about the vaccine, including the opinions of scientists and scientific organizations, resulted in lower vaccine refusal and hesitancy. In agreement, previous national studies have shown increased COVID-19 vaccine refusal rates among those with concerns about vaccine safety [21,22]. Additionally, a study conducted on people from two countries in the Eastern Mediterranean region with similar cultural backgrounds (Jordan and Kuwait) reached the same conclusion [35]. Misinformation and mistrust can endorse conspiracy beliefs that make people abstain from receiving COVID-19 vaccines [36]. This finding turns our attention to the urgent need of national health organizations to overcome people’s concerns and correct misinformation.

Interestingly, compared with Saudi citizens, non-Saudi residents were more willing to receive the vaccine. This finding could be partially explained by our results that showed higher vaccine refusal among the unemployed than the employed, and since the great majority of non-Saudi residents are employed, it could be expected that they have to go out to earn their living and, consequently, have a higher possibility of infection with COVID-19. Individuals with high perceived threat and risk appraisal were shown in previous research to be more willing to receive the COVID-19 vaccine [37]. Further, non-Saudi residents have been known to share houses and even rooms with each other, which increases their possibility of contracting the virus. The high perceived risk among non-Saudi residents, due to their need to work and share houses, may explain why they are more willing than Saudi citizens to receive the vaccine.

Of note, some limitations should be addressed. First, because of social distancing restrictions, we had to resort to an online survey. Online surveys can hide non-response bias since respondents might have different sociodemographic characteristics and, consequently, different vaccine refusal and hesitancy rates compared with non-respondents. For example, respondents to online surveys, in general, tend to be younger than non-respondents [38]. Young age in this study was associated with vaccine refusal and hesitancy; thus, the generalization of our results to the whole population should be made cautiously. Second, although we investigated most potential risk factors for COVID-19 vaccine refusal and hesitancy, we believe that other cultural and religious variables could have played a role in the participants’ decision on vaccination. A qualitative assessment of such variables is, therefore, highly warranted. Third, the knowledge scale used in this study was based on a previous study and not validated as a result of the limited number of studies on the topic when proposing the study, so the scale needs to be validated.

## 5. Conclusions

Our study shows that compared to previous national studies, it seems that COVID-19 vaccine refusal and hesitancy among the general population in Saudi Arabia is declining. Age, Saudi citizenship, lack of vaccine knowledge, and having concerns about the vaccine are major risk factors for COVID-19 vaccine refusal and hesitancy. We think that the national campaigns for COVID-19 vaccination should target young Saudi men.

## Figures and Tables

**Table 1:** Sociodemographic characteristics of the participants.

Characteristics		*N* = 1935 (%)
Sex	Men	925 (47.8)
	Women	1010 (52.2)
Age (mean ± SD)		36.6 ± 11.2
Education level	High school or lower	129 (6.7)
	Diploma	388 (20.1)
	University	1137 (58.8)
	Post-graduate	281 (14.4)
Nationality, %	Saudi	1226 (63.4)
	Non-Saudi	709 (36.6)
Income, %	SAR ≤ 6000	762 (39.4)
	SAR 6001–12,000	565 (29.2)
	SAR 12,001–18,000	316 (16.3)
	SAR 18,001–24,000	151 (7.8)
	SAR > 24,000	141 (7.3)
History of chronic diseases, %	Yes	277 (14.3)
	
History of allergies, %	Yes	211 (10.9)
	
Previous COVID-19 infection	Yes	304 (15.7)
	

**Table 2:** Knowledge of participants about COVID-19 vaccines.

Statement	Strongly Disagree	Disagree	Neutral	Agree	Strongly Agree
I know people who are not allowed to take COVID-19 vaccines	153 (7.9)	322 (16.6)	376 (19.5)	850 (43.9)	234 (12.1)
I know the possible side effects of COVID-19 vaccines	102 (5.3)	294 (15.2)	422 (21.8)	918 (47.4)	199 (10.3)
I know the steps that have been taken to test the efficacy of COVID-19 vaccines	113 (5.8)	330 (17.1)	515 (26.6)	836 (43.3)	139 (7.2)
I know the steps that have been taken to test the safety of COVID-19 vaccines	127 (6.6)	321 (16.6)	532 (27.5)	813 (42.0)	142 (7.3)
I know the opinion of scientists about COVID-19 vaccines	62 (3.2)	191 (9.9)	538 (27.8)	939 (48.5)	205 (10.6)
I know the opinion of trusted health organizations about COVID-19 vaccines	62 (3.2)	153 (7.9)	487 (25.2)	1021 (52.8)	212 (11.0)

**Table 3:** Concerns of participants about COVID-19 vaccines.

Statement	Strongly Disagree	Disagree	Neutral	Agree	Strongly Agree
COVID-19 vaccines have serious adverse effects	219 (11.3)	444 (22.9)	799 (41.3)	344 (17.8)	129 (6.7)
Drug companies are interested in COVID-19 vaccine sales only	209 (10.8)	548 (28.3)	669 (34.6)	358 (18.5)	151 (7.8)
The healthcare system is not trustworthy regarding COVID-19 vaccines	491 (25.4)	766 (39.6)	466 (24.0)	154 (8.0)	58 (3.0)
COVID-19 vaccines are ineffective	399 (20.7)	742 (38.3)	616 (31.8)	137 (7.1)	41 (2.1)
COVID-19 vaccines are not tested enough on people	202 (10.5)	498 (25.7)	540 (27.9)	499 (25.8)	196 (10.1)

**Table 4:** Multinomial logistic regression for predictors of refusal and hesitancy to uptake the COVID-19 vaccine.

Variables		Refusal		Hesitancy	
		OR	95% CI for OR	OR	95% CI for OR
Concerns		**1.503**	(1.423, 1.588)	**1.321**	(1.270, 1.374)
Knowledge		**0.937**	(0.898, 0.977)	**0.907**	(0.879, 0.936)
Age		**0.961**	(0.938, 0.984)	**0.977**	(0.961, 0.992)
Gender	Male	-	-	-	-
	Female	0.872	(0.585, 1.299)	1.133	(0.851, 1.508)
Marital Status	Married	-	-	-	-
	Not Married	0.617	(0.376, 1.014)	0.866	(0.612, 1.225)
Nationality	Saudi	-	-	-	-
	Non-Saudi	**0.182**	(0.105, 0.316)	**0.231**	(0.161, 0.333)
Education	Less than Bachelor	-	-	-	-
	Bachelor	1.076	(0.704, 1.644)	1.040	(0.766, 1.413)
	Graduate	0.834	(0.448, 1.550)	0.858	(0.549, 1.342)
Income	Less than SAR 6000	-	-	-	-
	SAR 6000 to 12,000	1.116	(0.674, 1.848)	**0.607**	(0.423, 0.871)
	SAR 12,001 to 18,000	0.978	(0.545, 1.757)	0.734	(0.475, 1.133)
	More Than SAR 18,000	**2.325**	(1.154, 4.682)	1.283	(0.781, 2.107)
Chronic	Yes	-	-	-	-
	No	**0.521**	(0.311, 0.873)	**0.640**	(0.440, 0.932)
Allergy	Yes	-	-	-	-
	No	0.722	(0.435, 1.196)	1.028	(0.689, 1.534)
Had COVID-19	Yes	-	-	-	-
	No	1.172	(0.708,1.939)	1.294	(0.898, 1.865)

Bold results indicate significant results.

## Data Availability

Data are available upon reasonable request by contacting the corresponding author.
